# A green approach to the synthesis of novel phytosphingolipidyl β-cyclodextrin designed to interact with membranes

**DOI:** 10.3762/bjoc.10.278

**Published:** 2014-11-12

**Authors:** Yong Miao, Florence Djedaïni-Pilard, Véronique Bonnet

**Affiliations:** 1 LG2A FRE-CNRS 3517 Institut de Chimie de Picardie FR CNRS 3085, SFR Condorcet, UPJV, 33 rue St Leu, 80039 Amiens, France

**Keywords:** fatty ester, green chemistry, lipase, mono-substituted amphiphilic cyclodextrins, peptide Coupling, solvent-free medium, transesterification

## Abstract

This work reports the synthesis of a new family of mono-substituted amphiphilic cyclodextrins using a green methodology. Reactions using greener and safer catalysts with more environmentally friendly purification solvents were performed. Four unreported mono-substituted cyclodextrins bearing a phytosphingolipidyl chain and a fatty acid chain (C_10_, C_12_, C_14_ and C_18_) were successfully obtained with a promising yield.

## Introduction

Cyclodextrins (CDs) are sustainable compounds which are particularly interesting in the frame of pharmaceutical and cosmetic applications and food industry thanks to their ability to encapsulate the hydrophobic molecules by forming inclusion complexes [1]. Moreover, the modified amphiphilic CDs are able to form nanoparticles without cosolvent or surfactant which could be ideal for drug delivery systems [2]. In order to have good water solubility, selectively modified (methylated) CDs are mostly desired [3]. Among the selectively modified CDs, mono-substituded CD derivatives do not only have self-assembly properties, but also have a good water solubility, allowing passive diffusion across a membrane through its lipid structures or through its water-filled pores or tight junctions [4]. Different types of hydrophobic moieties (3-methylcarboxydodecan-5-enoic acyl [5], cholesterol [6], phospholipids [7]) have been successfully selectively grafted on the C6 position of CD. However, new hydrophobic anchors are still desired to vary membrane interactions. We have thus discovered that the fatty acyl-permethylated-β-CD cannot form nanoparticles in aqueous solvent probably due to the single lipidic chain inside the CD cavity [4,8], and later we found that bicatenar compounds whose hydrophobic moiety is composed of two chains, can form nanoparticles giving low critical aggregation concentration (CAC) [9]. Moreover, to the best of our knowledge, only few green approaches were described to modify cyclodextrins (enzyme catalysis [8–9], green solvents [10]).

**Figure 1:**
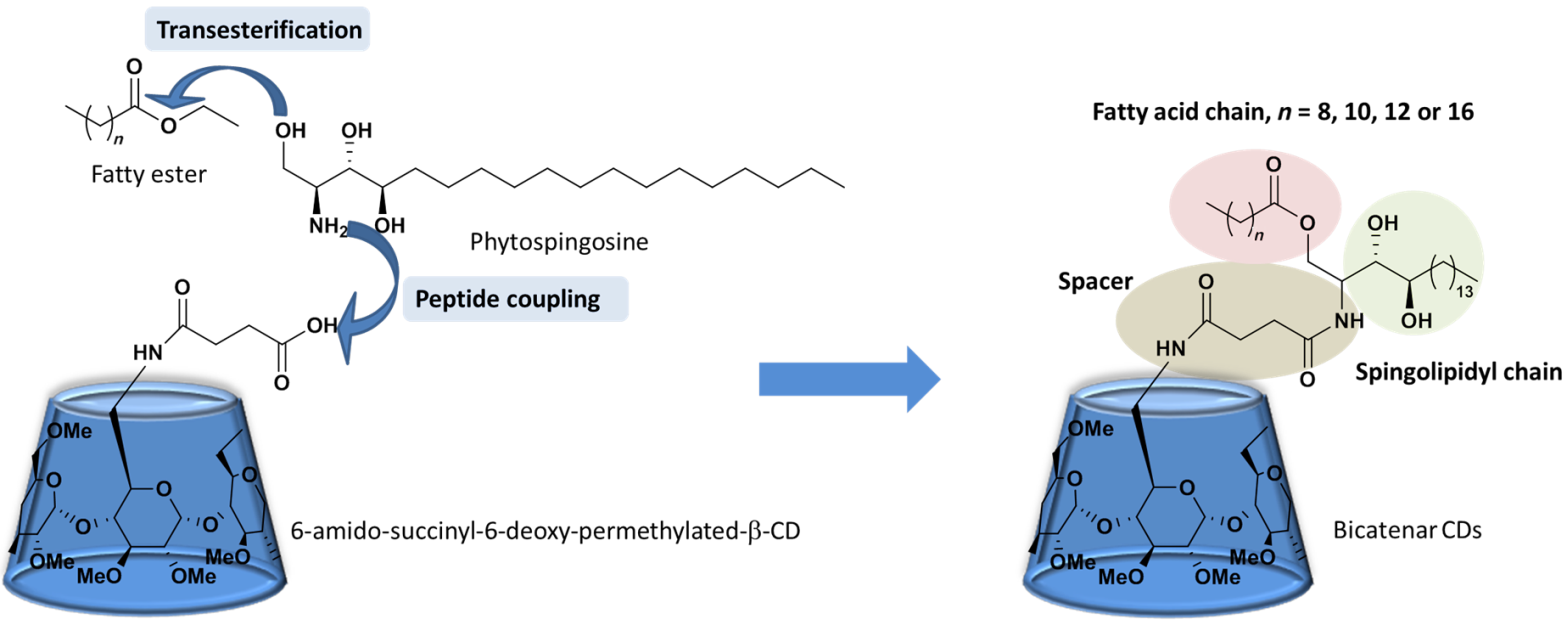
Targeted modified cyclodextrins.

The use of phytosphingosine as a sustainable hydrophobic compound bearing one primary amine, one primary alcohol and two secondary alcohols has attracted our interest (See Figure 1 ). Its amino group can be used to couple with 6-amidosuccinyl-6-desoxy-permethylated-β-CD, and thereafter, the primary alcohol can be used for the enzymatically catalyzed transesterification with the corresponding fatty ester, which may lead to bicatenar amphiphilic CDs. At the same time, the two remaining secondary alcohols might increase the solubility of the modified CD compounds.

The aim of this work is to synthesize new bicatenar CDs using green methods described for the first time in cyclodextrin chemistry via solvent-free substitution of aminocyclodextrin followed by the use of a new, more environmentally friendly peptide coupling agent known as COMU (1-cyano-2-ethoxy-2-oxoethylidenaminooxy)dimethylaminomorpholinocarbenium hexafluorophosphate). Finally, by optimized enzymatic transesterification, sphingolipid myelin analogues bearing CD as polar heads were synthesized to interact with ceramids of brain membranes.

## Results and Discussion

### Insertion of the spacer

**Scheme 1:**
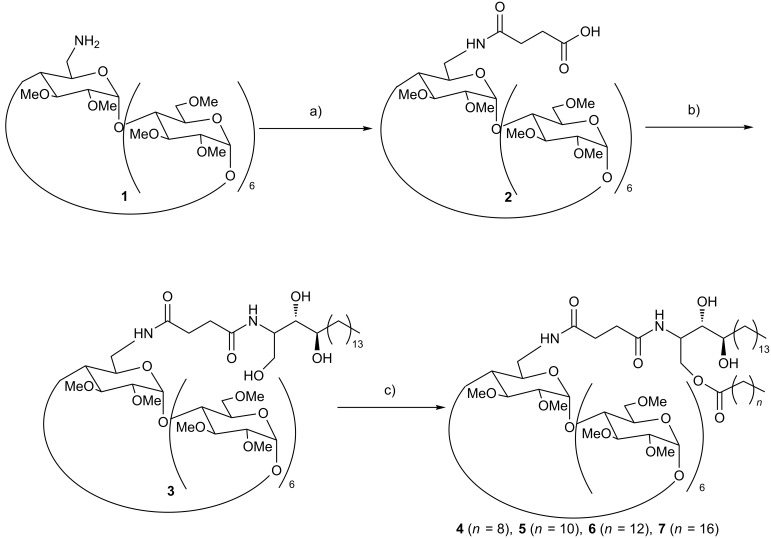
Synthesis of bicatenar CDs **4**, **5**, **6** and **7**; a) succinic anhydride, 135 °C, 10 min, 70%; b) phytosphingosine, COMU, anhydrous DMF, 24 h, 80%; c) for **4**, Lipozyme^®^, ethyl decanoate, 50 °C, 8 h, 74%; for **5**, ethyl dodecanoate, 50 °C, 8 h, 68%; for **6**, ethyl myristate, 50 °C, 12 h, 64%; For **7**, ethyl stearate, 50 °C, 12 h, 60%.

A procedure for the preparation of compound **1** has already been described in previous articles [8]. The reaction was conducted in freshly distilled DMF using 1 equivalent of 6-amino-6-deoxy-permethylated-β-CD and 3 equivalents of succinic anhydride at room temperature for 24 hours, leading to yields above 90% (see Scheme 1). However it is known that DMF can cause cancer in humans, thus it was decided to conduct this reaction without DMF. Recently, Zinck et al. have reported the controlled ring-opening polymerization of lactide using bulk conditions [11]. They demonstrated that the reaction in bulk is much faster than the reaction in dichloromethane (for a ratio monomer/ROH of 30, 25 min, conversion = 95% in bulk vs 39 h, conversion = 46% in dichloromethane). Inspired by the work of Zinck et al., the reaction was performed using similar conditions. A determined amount of succinic anhydride was firstly melted at 135 °C (superior to its melting point temperature), and then CD amine was added to the melted medium. The mixture was left to react for 10 minutes. The use of TLC indicated the end of the reaction. After the purification, yields of above 70% were obtained. It was indeed observed that solvent-free conditions can be employed in this reaction. The reaction is much faster without solvent due to the high reactant concentration and high temperatures used. The yield of the reaction performed in solvent-free condition is slightly lower than those obtained when conducted in DMF. This may be linked to the purification, since a large quantity of succinic anhydride was used in the reaction. Excess succinic anhydride can be recovered easily by filtration at low temperature but part of the compound underwent methanolysis, preventing its reuse for another reaction.

### Insertion of phytosphingolipidyl arm

In this step, the peptide coupling reagent plays an important role. Different peptide coupling reagents have been applied in this reaction, among which DIC/HOBt is one of the most used. In previous work, the peptide coupling reaction between compound **1** and ethanolamine using DIC/HOBt as the catalyst has been reported [8]. Even when large quantities of DIC and HOBt were used (30 equiv DIC and 30 equiv HOBt), only modest yields were obtained (48%). HATU (*O*-(7-azabenzotriazol-1-yl)-*N,N,N’,N’*-tetramethyluronium hexafluorophosphate) has also been employed in a similar coupling peptide reaction. Promising results were obtained (yield = 90%) using only 1.5 equivalents HATU [9]. It is noticeable that HATU is a flammable and costly compound which may cause accidents in industrial production and thus increase the price of the final product. In view of the above problems, a safer, less costly and more efficient peptide coupling reagent is desired. Recently, COMU, a new uronium salt derived from Oxyma (ethyl (hydroxyimino)cyanoacetate), has been reported as a greener, safer and more efficient peptide coupling reagent [12]. It was decided that COMU would be used as a peptide coupling reagent in the modification of cyclodextrin using the described conditions in previous studies [9]. The carboxyl group of compound **2** was activated with 2 equivalents of COMU in freshly distilled DMF during 1 hour at room temperature, then 10 equivalents of phytosphingosine was added to the reaction. After 24 hours, TLC indicated that the reaction was finished. After purification, very promising results were obtained (yield = 80%, see Scheme 1). Indeed, COMU is an efficient peptide coupling reagent and can be used in the synthesis of cyclodextrin derivatives.

### Insertion of fatty acid chain

In previous work, the synthesis of the reaction of permethylated 6-amino-6-deoxy-β-cyclodextrin and vinyl ester catalyzed by various lipases in solvent free medium was reported [13]. Using specific experimental conditions such as an open reactor leads to a better yield [9,14], however, the reaction time is still long for the synthesis of bicatenar glycerolipidyl derivatives (5 days, 54% for the reaction using ethyl stearate). Since the boiling point of the co-solvent (long chain fatty ester) is relatively high and the byproduct ethanol can be easily eliminated, a vacuum system can be applied in order to accelerate the reaction. A rotary evaporator allows both the reaction to be conducted under stirring whilst under vacuum at a controlled temperature. So it was decided to perform the transesterification of 100 mg of **3** in 2 mL of fatty ester catalyzed by 100 mg of Lipozyme^®^ using a rotary evaporator at 50 °C and 90 mbar. TLC indicated that after only 8 hours, the transesterification of ethyl decanoate with product **3** was complete. Column chromatography in ethyl acetate/MeOH (from 100:0 to 80:20) was performed on crude to recover fatty esters as a pure compound which could be reused after evaporation of solvents and modified cyclodextrin **4** with good yield of 74%. Other reactions with longer chains were also successful with promising yields (from 60% to 68% in 8–14 h). Compounds **4**–**7** were fully characterized by NMR and MS. MS data showed that **3** was totally removed from final esters and shift in ^13^C NMR of modified CH_2_O proved the regioselectivity of the reaction (see Supporting Information File 1).

Surprisingly, the four new compounds, unlike the other previously reported bicatenar CDs which are soluble in aqueous solution [9], have a very limited solubility in water (<50 µM), so that the CAC could not be determined. The reason why these compounds have such a low solubility is still unclear especially as the monocatenary compound **3** has a poor solubility in water too (<0.1 mM). It may be due to the formation of the H-bonds between hydroxy groups of the sphingolipidyl chains. However, more studies will be performed in the future.

## Conclusion

Four new phytosphingolipidyl CDs have been successfully synthesized using green methods and safer, less costly reagents. These changes may reduce the price of the final products and decrease the risk in industrial production.

## Supporting Information

File 1 Experimental and analytical data.
