# Supplementary material for: A green approach to the synthesis of novel phytosphingolipidyl β-cyclodextrin designed to interact with membranes
Source: Beilstein J Org Chem. 2014 Nov 12;10:2654–7. doi: 10.3762/bjoc.10.278 (PMC4273305; doi:10.3762/bjoc.10.278)
Supplement: File 1 — Experimental and analytical data. [file Beilstein_J_Org_Chem-10-2654-s001.pdf]

Supporting Information

for

A green approach to the synthesis of novel phytosphingolipidyl β-cyclodextrin designed to interact with membranes

Yong Miao, Florence Djedaïni-Pilard and Véronique Bonnet*

Address: LG2A FRE-CNRS 3517 Institut de Chimie de Picardie FR CNRS 3085, SFR Condorcet, UPJV, 33 rue St Leu 80039 Amiens France

Email: Véronique Bonnet* - veronique.bonnet@u-picardie.fr
*Corresponding author

Experimental and analytical data

Experimental

Materials and methods

Native β-cyclodextrin was obtained from Wacker Chemicals (Germany). Ethyl decanoate, ethyl laurate, ethyl myristate, ethyl stearate and COMU were purchased from Sigma-Aldrich. Lipase immobilized from *Mucor miehei,* Lipozyme®*,* was purchased from Fluka Chemie GmbH (Germany). Phytosphingosine was purchased from TCI. Other chemicals were purchased from Sigma-Aldrich. All the solvents

employed for the reactions were distilled once before use. Deuterated solvents were purchased from Euriotop (France).

Stepwise control of the reactions has been readily achieved using ESI-MS in the positive ion mode using a ZQ 4000 quadrupole mass spectrometer (Waters-Micromass, Manchester, UK). Reaction media were diluted in MeOH (0.001 mg/ml) and the solutions were filtered before introducing. In the same conditions, the structure elucidation of the final products was further confirmed by High-Resolution Mass Spectrometry (ESI-HRMS) using electrospray infusion mode performed in positive mode on a QTOF Ultima Global instrument (Waters-Micromass, Manchester, UK). An external calibration was done. Data acquisition and processing were performed with MASS LYNX 4.0 software. ^{1}H NMR and ^{13}C NMR spectra were recorded with a Bruker AVANCE DPX 300 at 300.16 and 75.78 MHz, respectively, and a Bruker AVANCE DRX 600 spectrometer at 600 and 150.1 MHz, respectively, in deuterated chloroform ($CDCl_3$) at 25 °C. All compounds were characterized by 1H-1H (COSY) and 1H-13C (HSQC) correlation experiments. Chemical shifts are given in δ-units measured downfield from Me_4Si at 0 ppm using the residual solvent signal as secondary reference.

6^{I}-Amino-6^{I}-deoxy-2^{I},3^{I}-di-*O*-methyl-hexakis(2$^{II\text{-}VII}$, 3$^{II\text{-}VII}$, 6$^{II\text{-}VII}$ -tri-*O*-methyl)cyclomaltoheptaose (1)

1 was obtained in four steps from 10 g of native cyclodextrin, as described in the literature [1]. It was purified by column chromatography on silica gel (CH_2Cl_2/MeOH 9/1 (v/v), R_f = 0.5), giving an overall yield of 30%. The analytical data are in good agreement with the literature [1].

6^{I}-Succinylamino-6^{I}-deoxy-2^{I},3^{I}-di-*O*-methyl-hexakis(2^{II-VII}, 3^{II-VII}, 6^{II-VII} -tri-*O*-methyl)cyclomaltoheptaose (2)

1 g (10 mmol, 47 eq.) of succinic anhydride was introduced to a flask of 10 ml, and the medium was stirred magnetically at 135°C until the solid was completely melted (about 5 minutes). Then 300 mg of product **1** (212 µmol, 1 eq.) was added to the melt medium, and the reaction was stirred magnetically for 10 minutes. The reaction was cooled down to room temperature, and 5 ml of MeOH was added, ultrasonic cleaner was employed to break down the solid. Then mixture was filtered, and the solution was concentrated under vacuum. The crude was purified on silica gel (CH_2Cl_2/MeOH 9/1 (v/v), Rf = 0.5), giving 220 mg (yield = 70%). The analytical data are in good agreement with the literature [2].

6-(*N*-(3*S*,4*R*)-1,3,4-trihydroxyoctadecan-2-yl)succinylamido-6^{I}-deoxy-2^{I},3^{I}-di-*O*-methyl-hexakis(2^{II-VII}, 3^{II-VII}, 6^{II-VII} -tri-*O*-methyl)cyclomaltoheptaose (3)

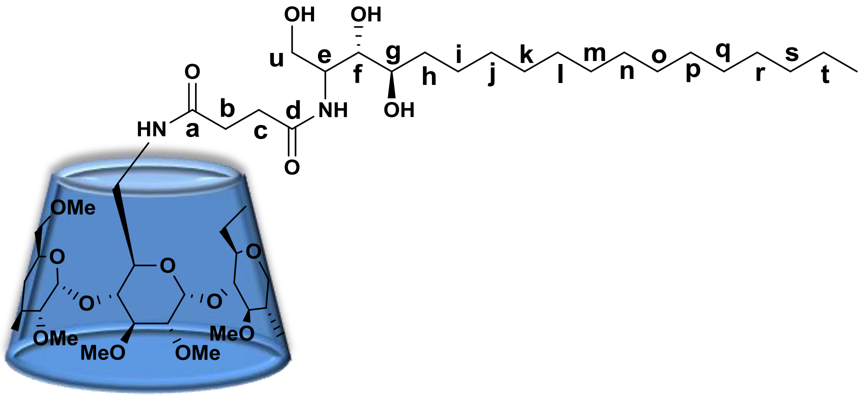

To a solution of 100 mg of **2** (66 µmol, 1eq) in 30 ml of fresh distilled DMF, 57 mg of COMU (133 µmol, 2 eq.) was added. The reaction was stirred magnetically at room temperature under argon for 1 hour. Then, 209 mg of phytosphingosine (660 µmol, 10 eq.) in 10 ml of DMF was added to the reaction. The mixture was stirred magnetically at room temperature under argon for 24 hours. The crude was purified

on silica gel (from pure EtOAc to EtOAc/MeOH 7/3(v/v)), giving 73 mg of solid (yield = 61%).

^{1}H-NMR (300 MHz, $CDCl_3$) δ (ppm) 7.1 (s, 2H, 2NHCO), 5.3-5.0 (m, 7H, $H^{I\text{-}VII}_{1CD}$), 4.4-3.0 (m, 50H, $H^{I\text{-}VII}_{2CD}$, $H^{I\text{-}VII}_{3CD,}$ $H^{I\text{-}VII}_{4CD,}$ $H^{I\text{-}VII}_{5CD,}$ $H^{I\text{-}VII}_{6CD}$ + $OH_{v,w,x}$ + $CH_{e,f,g}$ + C_uH_2), 3.6 (s, 18H, $O_{6CD}CH_3$), 3.5 (s, 21H, $O_{3CD}CH_3$), 3.4 (s, 21H, $O_{2CD}CH_3$), 2.6-2.4 (m, 4H, C_bH_2 and C_cH_2), 0.9 (t, 3H, CH_3, J = 7Hz)

^{13}C-NMR (75 MHz, $CDCl_3$) δ (ppm) 173.30 (2C, $C(=O)_{a,d}$), 99.25 (7C, $C^{I\text{-}VII}_{1CD}$), 82.20-80.46 (28C, $C^{I\text{-}VII}_{2CD}$, $C^{I\text{-}VII}_{3CD,}$ $C^{I\text{-}VII}_{4CD,}$ $C^{I\text{-}VII}_{5CD}$), 71.20 (8C, $C^{I\text{-}VII}_{6CD,}$ $C_{f,g}H$), 61.40 (1C, C_uH_2), 58.90 (21C, $O_{2CD}CH_3$, $O_{3CD}CH_{3,}$ $O_{6CD}CH_{3,}$ C_eH), 40.00 (1C, C^{I}_{6CD}), 33.60 (1C, C_hH_2), 30.40 (2C, $C_{b\text{-}c}H_2$), 29.40 (10C, $C_{j\text{-}s}H_2$), 25.90 (1C, C_iH_2), 22.70 (1C, C_tH_2), 14.10 (1C, CH_3)

MS for $C_{84}H_{152}N_2O_{39}Na$ calcd 1835.9899, found 1835.9869

General procedure for the synthesis of compound 4, 5, 6 and 7

To a solution of 100 mg of compound **3** (55 µmol, 1 equiv) in 2 ml of corresponding fatty ester (excess), 100 mg of Lipozyme® was added. The reaction was set under rotary evaporator at 50 °C during 8 to 14 hours depending on the corresponding fatty ester. Lipozyme was filtered, and the solution was purified directly on silica gel (from pure EtOAc to EtOAc/MeOH 4:1(v/v)), giving white solid. Yields: **4** (80mg): 74%, **5** (75mg): 68%, **6** (72mg): 64%, **7** (69mg): 60%.

6-(*N*-(1-decanoyloxy-(3*S*,4*R*)-dihydroxyoctadecan-2-yl)succinamido-6^{I}-deoxy-2^{I},3^{I}-di-*O*-methyl-hexakis(2^{II-VII}, 3^{II-VII}, 6^{II-VII}-tri-*O*-methyl)cyclomaltoheptaose (4)

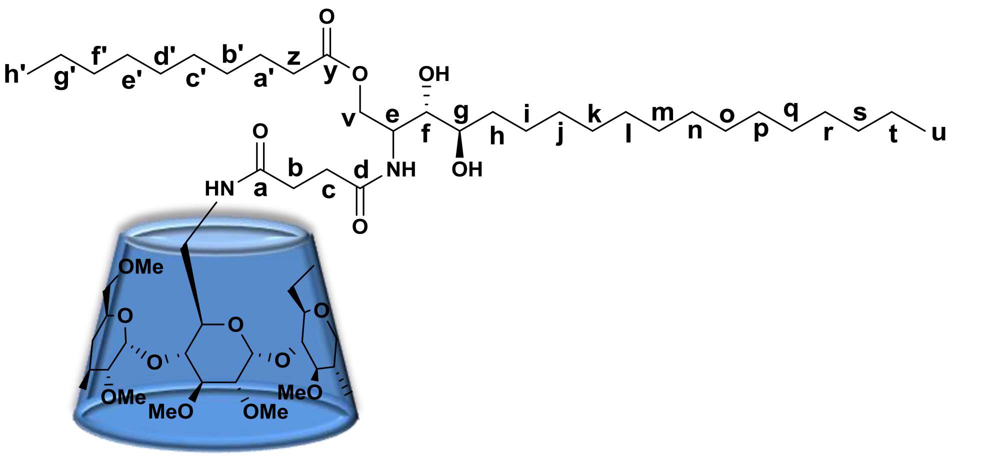

^{1}H-NMR (600 MHz, $CDCl_3$) δ (ppm) 6.55 (d, 1H, $N_{(d-e)}$Hco, J = 6 Hz), 6.39 (t, 1H, N_aHco, J = 6 Hz), 5.11 (m, 7H, H^{I-VII}_{1CD}), 4.29-4.36 (m, 3H, C_vH_2 and C_eH), 3.80 (m, 14H, H^{I-VII}_{6CD}), 3.32-3.68 (m, 83H, H^{I-VII}_{3CD}, H^{I-VII}_{4CD}, H^{I-VII}_{5CD}, $O_{6CD}CH_3$, $O_{3CD}CH_3$, $O_{2CD}CH_3$, $CH_{f,g}$), 3.17 (m, 7H, H^{I-VII}_{2CD}), 2.54-2.64 (m, 2H, C_cH_2), 2.41 (m, 2H, C_bH_2), 2.29 (t, 2H, C_zH_2, J = 7.51 Hz), 1.67 (m, 1H, C_hH^a), 1.59 (m, 2H, $C_{a'}H_2$), 1.51 (m, 1H, C_iH^a), 1.37 (m, 1H, C_hH^b), 1.31 (m, 1H, C_iH^b), 1.24 (m, 34H, $C_{j-t}H_2$, $C_{b'-g'}H_2$), 0.86 (t, 6H, C_uH_3 and $C_{h'}H_3$, J = 7.0 Hz)

^{13}C-NMR (150 MHz, $CDCl_3$) δ (ppm) 174.43, 172.58, 172.52 (3C, $C(=O)_{a,d,y}$), 99.23-98.68 (7C, C^{I-VII}_{1CD}), 82.27-79.73 (28C, C^{I-VII}_{2CD}, C^{I-VII}_{3CD}, C^{I-VII}_{4CD}, C^{I-VII}_{5CD}), 71.20 (8C, C^{I-VII}_{6CD}, $C_{f,g}H$), 63.08 (1C, C_vH_2), 61.69-58.45 (20C, $O_{2CD}CH_3$, $O_{3CD}CH_3$, $O_{6CD}CH_3$), 51.78 (1C, C_eH), 40.21 (1C, C^{I}_{6CD}), 34.38 (1C, C_zH_2), 33.63 (1C, C_hH_2), 31.98, 32.05 (2C, C_sH_2, $C_{f'}H_2$), 31.57,31.89 (2C, $C_{b-c}H_2$), 29.41 (13C, $C_{j-r}H_2$, $C_{b'-e'}H_2$), 26.15 (1C, C_iH_2), 25.02 (1C, $C_{a'}H_2$), 22.79, 22.81 (2C, C_tH_2, $C_{g'}H_2$), 14.25, 14.23 (2C, $C_{h'}H_3$, C_uH_3)

MS for $C_{94}H_{170}N_2O_{40}Na$ calcd 1990.1228 found 1990.1191

6-(*N*-(1-dodecanoyloxy-(3*S*,4*R*)-dihydroxyoctadecan-2-yl)succinamido-6^{I}-deoxy-2^{I},3^{I}-di-*O*-methyl-hexakis(2^{II-VII}, 3^{II-VII}, 6^{II-VII} -tri-*O*-methyl)cyclomaltoheptaose (5)

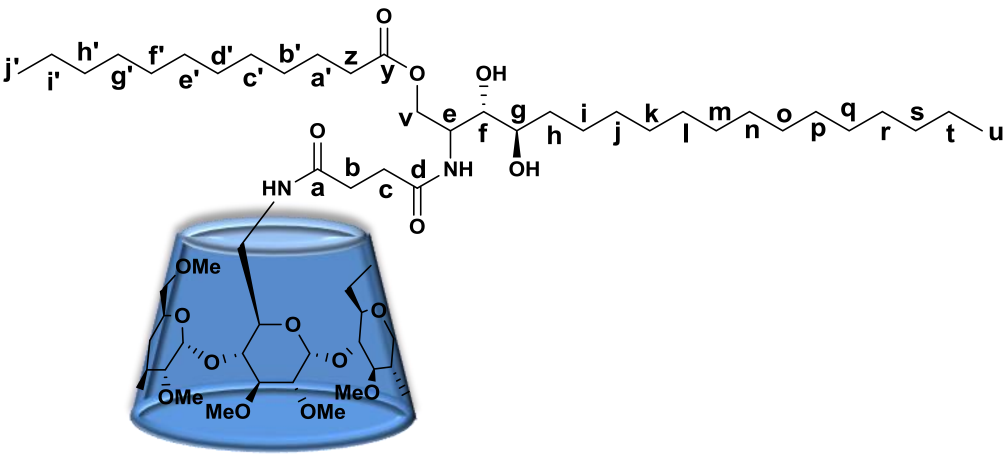

^{1}H-NMR (600 MHz, $CDCl_3$) δ (ppm) 6.55 (d, 1H, $N_{(d-e)}$Hco, J = 6 Hz), 6.39 (t, 1H, N_aHco, J = 6 Hz), 5.11 (m, 7H, H^{I-VII}_{1CD}), 4.29-4.36 (m, 3H, C_vH_2 and C_eH), 3.80 (m, 14H, H^{I-VII}_{6CD}), 3.32-3.68 (m, 83H, H^{I-VII}_{3CD}, H^{I-VII}_{4CD}, H^{I-VII}_{5CD}, $O_{6CD}CH_3$, $O_{3CD}CH_3$, $O_{2CD}CH_3$, $CH_{f,g}$), 3.17 (m, 7H, H^{I-VII}_{2CD}), 2.54-2.64 (m, 2H, C_cH_2), 2.41 (m, 2H, C_bH_2), 2.29 (t, 2H, C_zH_2, J = 7.51 Hz), 1.67 (m, 1H, C_hH^a), 1.59 (m, 2H, $C_{a'}H_2$), 1.51 (m, 1H, C_iH^a), 1.37 (m, 1H, C_hH^b), 1.31 (m, 1H, C_iH^b), 1.24 (m, 38H, $C_{j-t}H_2$, $C_{b'-i'}H_2$), 0.86 (t, 6H, C_uH_3 and $C_{h'}H_3$, J = 7.0 Hz)

^{13}C-NMR (150 MHz, $CDCl_3$) δ (ppm) 174.39, 172.55, 172.52 (3C, $C(=O)_{a,d,y}$), 99.17-98.62 (7C, C^{I-VII}_{1CD}), 82.27-79.59 (28C, C^{I-VII}_{2CD}, C^{I-VII}_{3CD}, C^{I-VII}_{4CD}, C^{I-VII}_{5CD}), 71.20 (8C, C^{I-VII}_{6CD}, $C_{f,g}H$), 63.00 (1C, C_vH_2), 61.63-58.40 (20C, $O_{2CD}CH_3$, $O_{3CD}CH_3$, $O_{6CD}CH_3$), 51.62 (1C, C_eH), 40.17 (1C, C^{I}_{6CD}), 34.33 (1C, C_zH_2), 33.62 (1C, C_hH_2), 31.98, 32.00 (2C, C_sH_2, $C_{h'}H_2$), 31.50,31.78 (2C, $C_{b-c}H_2$), 29.41 (15C, $C_{j-r}H_2$, $C_{b'-g'}H_2$), 26.15 (1C, C_iH_2), 24.79 (1C, $C_{a'}H_2$), 22.76 (2C, C_tH_2, $C_{i'}H_2$), 14.20 (2C, $C_{j'}H_3$, C_uH_3)

MS for $C_{96}H_{174}N_2O_{40}Na$ calcd 2018.1541, found 2018.1521

6-(*N*-(1-myristoyloxy-(3*S*,4*R*)-dihydroxyoctadecan-2-yl)succinamido-6^{I}-deoxy-2^{I},3^{I}-di-*O*-methyl-hexakis(2^{II-VII}, 3^{II-VII}, 6^{II-VII} -tri-*O*-methyl)cyclomaltoheptaose (6)

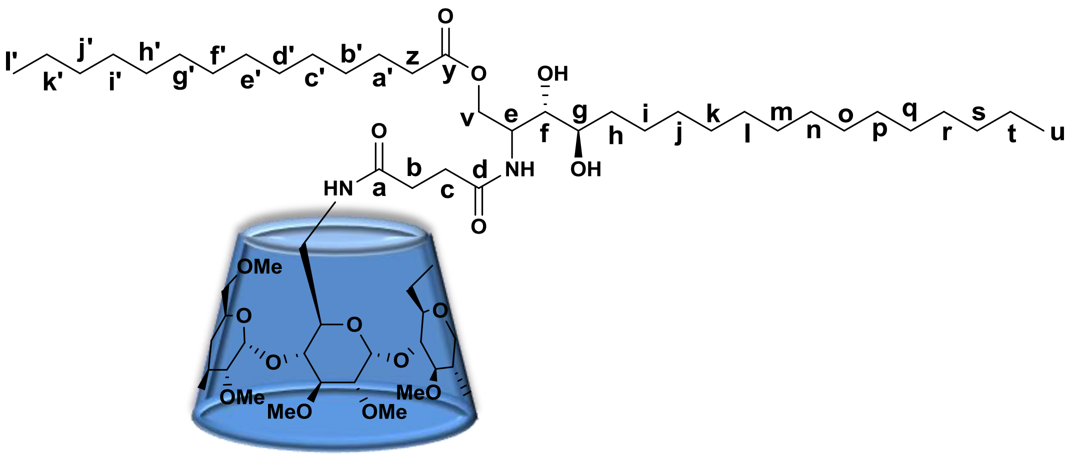

^{1}H-NMR (600 MHz, CDCl$_3$) δ (ppm) 6.55 (d, 1H, N$_{(d-e)}$Hco, J = 6 Hz), 6.39 (t, 1H, N$_a$Hco, J = 6 Hz), 5.11 (m, 7H, H$^{I-VII}_{1CD}$), 4.29-4.36 (m, 3H, C$_v$H$_2$ and C$_e$H), 3.80 (m, 14H, H$^{I-VII}_{6CD}$), 3.32-3.68 (m, 83H, H$^{I-VII}_{3CD,}$ H$^{I-VII}_{4CD,}$ H$^{I-VII}_{5CD,}$ *O*$_{6CD}$CH$_{3,}$ *O*$_{3CD}$CH$_{3,}$ *O*$_{2CD}$CH$_{3,}$ CH$_{f,g}$), 3.17 (m, 7H, H$^{I-VII}_{2CD}$), 2.54-2.64 (m, 2H, C$_c$H$_2$), 2.41 (m, 2H, C$_b$H$_2$), 2.29 (t, 2H, C$_z$H$_{2,}$ J = 7.51 Hz), 1.67 (m, 1H, C$_h$H^a), 1.59 (m, 2H, C$_{a'}$H$_2$), 1.51 (m, 1H, C$_i$H^a), 1.37 (m, 1H, C$_h$H^b), 1.31 (m, 1H, C$_i$H^b), 1.24 (m, 42H, C$_{j-t}$H$_2$, C$_{b'-k'}$H$_2$), 0.86 (t, 6H, C$_u$H$_3$ and C$_{h'}$H$_3$, J = 7.0 Hz)

^{13}C-NMR (150 MHz, CDCl$_3$) δ (ppm) 174.40, 172.57, 172.50 (3C, C(=O)$_{a,d,y}$), 99.23-98.82 (7C, C$^{I-VII}_{1CD}$), 82.26-79.72 (28C, C$^{I-VII}_{2CD,}$ C$^{I-VII}_{3CD,}$ C$^{I-VII}_{4CD,}$ C$^{I-VII}_{5CD}$), 70.00-74.50 (8C, C$^{I-VII}_{6CD,}$ C$_{f,g}$H), 63.10 (1C, C$_v$H$_2$), 61.69-58.43 (20C, *O*$_{2CD}$CH$_3$, *O*$_{3CD}$CH$_3$, *O*$_{6CD}$CH$_3$), 51.76 (1C, C$_e$H), 40.22 (1C, C$^{I}_{6CD}$), 34.37 (1C, C$_z$H$_2$), 33.58 (1C, C$_h$H$_2$), 32.04 (2C, C$_s$H$_{2,}$ C$_{j'}$H$_2$), 31.60,31.91 (2C, C$_{b-c}$H$_2$), 29.23-29.92 (17C, C$_{j-r}$H$_{2,}$ C$_{b'-i'}$H$_2$), 26.14 (1C, C$_i$H$_2$), 25.02 (1C, C$_{a'}$H$_2$), 22.81 (2C, C$_t$H$_{2,}$ C$_{k'}$H$_2$), 14.20,14.38 (2C, C$_{l'}$H$_{3,}$ C$_u$H$_3$)

MS for C$_{98}$H$_{178}$N$_2$O$_{40}$Na calcd 2046.1854, found 2046.1753

6-(*N*-(1-octadecanoyloxy-(3*S*-4*R*)-dihydroxyoctadecan-2-yl)succinamido)-6^{I}-deoxy-2^{I},3^{I}-di-*O*-methyl-hexakis(2^{II-VII},3^{II-VII},6^{II-VII}-tri-*O*-methyl)cyclomaltoheptaose (7)

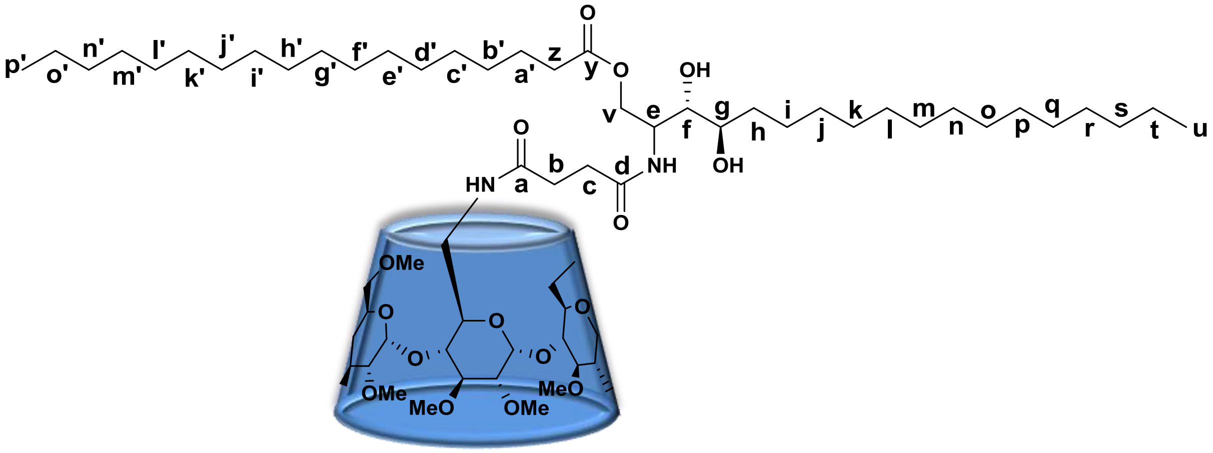

^{1}H-NMR (600 MHz, $CDCl_3$) δ (ppm) 6.55 (d, 1H, $N_{(d-e)}$Hco, J = 6 Hz), 6.39 (t, 1H, N_aHco, J = 6 Hz), 5.11 (m, 7H, H^{I-VII}_{1CD}), 4.29-4.36 (m, 3H, C_vH_2 and C_eH), 3.80 (m, 14H, H^{I-VII}_{6CD}), 3.32-3.68 (m, 83H, H^{I-VII}_{3CD}, H^{I-VII}_{4CD}, H^{I-VII}_{5CD}, $O_{6CD}CH_3$, $O_{3CD}CH_3$, $O_{2CD}CH_3$, $CH_{f,g}$), 3.17 (m, 7H, H^{I-VII}_{2CD}), 2.54-2.64 (m, 2H, C_cH_2), 2.41 (m, 2H, C_bH_2), 2.29 (t, 2H, C_zH_2, J = 7.51 Hz), 1.67 (m, 1H, C_hH^a), 1.59 (m, 2H, $C_{a'}H_2$), 1.51 (m, 1H, C_iH^a), 1.37 (m, 1H, C_hH^b), 1.31 (m, 1H, C_iH^b), 1.24 (m, 50H, $C_{j-t}H_2$, $C_{b'-o'}H_2$), 0.86 (t, 6H, C_uH_3 and $C_{h'}H_3$, J = 7.0 Hz)

^{13}C-NMR (150 MHz, $CDCl_3$) δ (ppm) 174.40, 172.63, 172.54 (3C, $C(=O)_{a,d,y}$), 99.23-98.67 (7C, C^{I-VII}_{1CD}), 82.26-79.72 (28C, C^{I-VII}_{2CD}, C^{I-VII}_{3CD}, C^{I-VII}_{4CD}, C^{I-VII}_{5CD}), 70.00-74.50 (8C, C^{I-VII}_{6CD}, $C_{f,g}H$), 63.13 (1C, C_vH_2), 61.69-58.43 (20C, $O_{2CD}CH_3$, $O_{3CD}CH_3$, $O_{6CD}CH_3$), 51.76 (1C, C_eH), 40.24 (1C, C^{I}_{6CD}), 34.36 (1C, C_zH_2), 33.52 (1C, C_hH_2), 32.04 (2C, C_sH_2, $C_{n'}H_2$), 31.60,31.91 (2C, $C_{b-c}H_2$), 29.23-29.92 (21C, $C_{j-r}H_2$, $C_{b'-m'}H_2$), 26.14 (1C, C_iH_2), 25.02 (1C, $C_{a'}H_2$), 22.81 (2C, C_tH_2, $C_{o'}H_2$), 14.24 (2C, $C_{p'}H_3$, C_uH_3)

MS for $C_{102}H_{186}N_2O_{40}Na$ calcd 2102.2480, found 2102.2424

References

1. Djedaini-Pilard, F.; Azaroual-Bellanger, N.; Gosnat, M.; Vernet, D.; Perly, B. *J. Chem. Soc., Perkin Trans. 2,* **1995**, 723-730.

2. Angelova, A.; Fajolles, C.; Hocquelet, C.; Djedaïni-Pilard, F.; Lesieur, S.; Bonnet, V.; Perly, B.; Lebas, G.; Mauclaire, L. *J. Colloid Interface Sci.,* **2008,** *322*, 304-314.

^{1}H NMR, ^{13}C NMR and HRMS spectra of compounds 3, 4, 5, 6 and 7

Compound 3

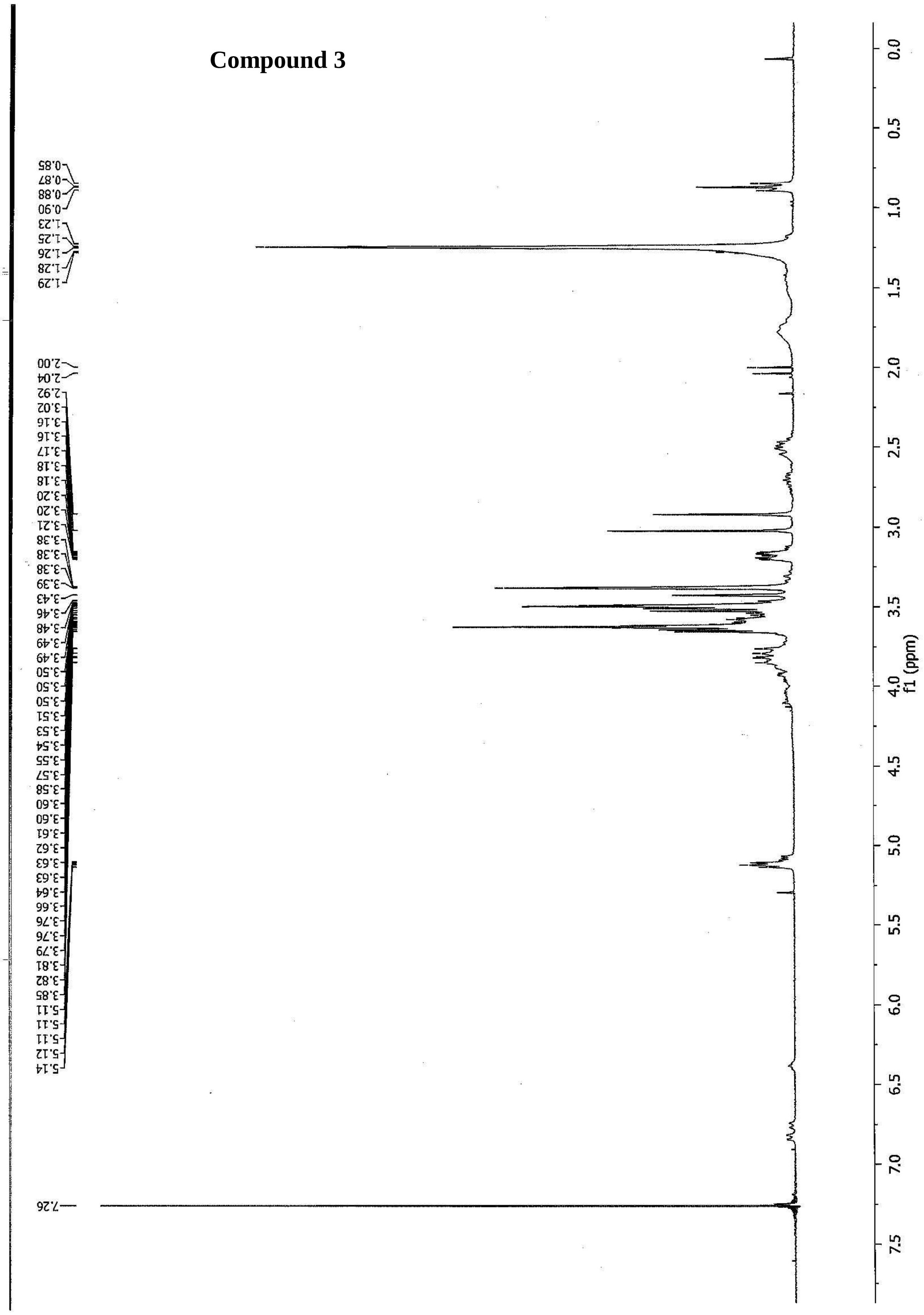
0.85
0.87
0.88
0.90
1.23
1.25
1.26
1.28
1.29
2.00
2.04
2.92
3.02
3.16
3.16
3.17
3.18
3.18
3.20
3.20
3.21
3.38
3.38
3.38
3.39
3.43
3.46
3.48
3.49
3.49
3.50
3.50
3.50
3.51
3.53
3.54
3.55
3.57
3.58
3.60
3.60
3.61
3.62
3.63
3.63
3.64
3.66
3.76
3.76
3.79
3.81
3.82
3.85
5.11
5.11
5.11
5.12
5.14
7.26
0.0
0.5
1.0
1.5
2.0
2.5
3.0
3.5
4.0
4.5
5.0
5.5
6.0
6.5
7.0
7.5
f1 (ppm)

Compound 3

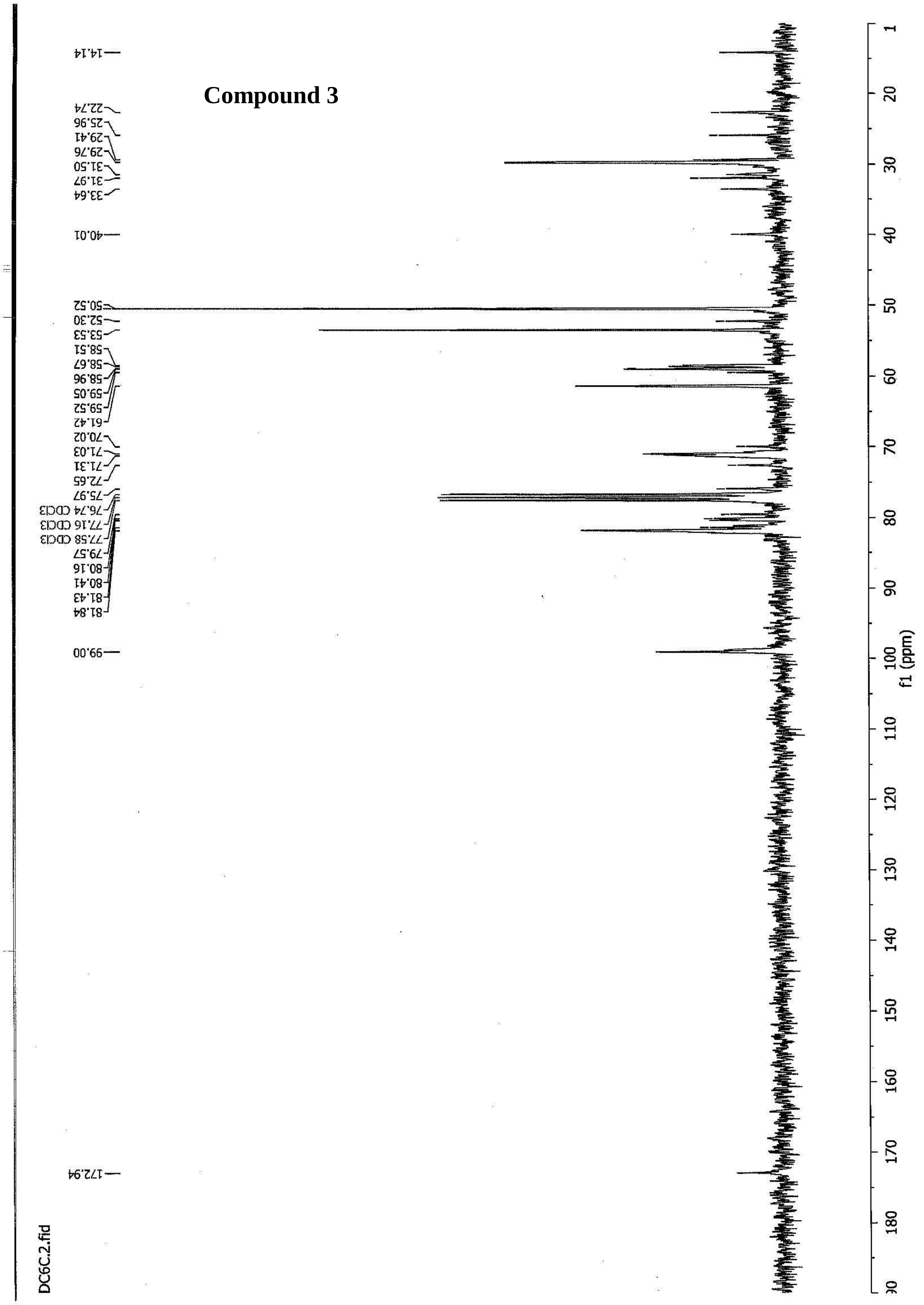
14.14
22.74
25.96
29.41
29.76
31.50
31.97
33.64
40.01
50.52
52.30
53.53
58.51
58.67
58.96
59.05
59.52
61.42
70.02
71.03
71.31
72.65
75.97
76.74 CDCl3
77.16 CDCl3
77.58 CDCl3
79.57
80.16
80.41
81.43
81.84
99.00
172.94
DC6C.2.fid
f1 (ppm)

Compound 3

Elemental Composition Report **Page 1**

Single Mass Analysis
Tolerance = 5.0 PPM / DBE: min = -1.5, max = 150.0
Isotope cluster parameters: Separation = 1.0 Abundance = 1.0%

Monoisotopic Mass, Even Electron Ions
2567 formula(e) evaluated with 4 results within limits (up to 50 closest results for each mass)

25-Jun-2010
VB DC6D-LOCK 12 (0.311) AM (Cen,5, 80.00, Ar,0.0,294.94,0.00,LS 5); Sm (SG, 2x3.00); Cm (10:12) 1: TOF MS ES+ 1.20e3

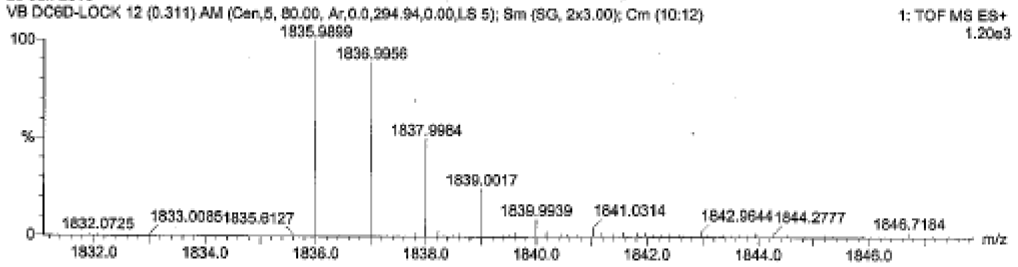

Mass	Calc. Mass	mDa	PPM	DBE	Score	Formula
Minimum:				-1.5		
Maximum:		2.0	5.0	150.0		
1835.9899	1835.9907	-0.8	-0.4	73.5	3	C84 H28 N7 O40 Na K
	1835.9888	1.1	0.6	5.5	2	C84 H157 O38 Na K
	1835.9925	-2.6	-1.4	69.5	4	C84 H33 N5 O39 Na K2
	1835.9870	2.9	1.6	9.5	1	C84 H152 N2 O39 Na

Product 4 ^{1}H NMR

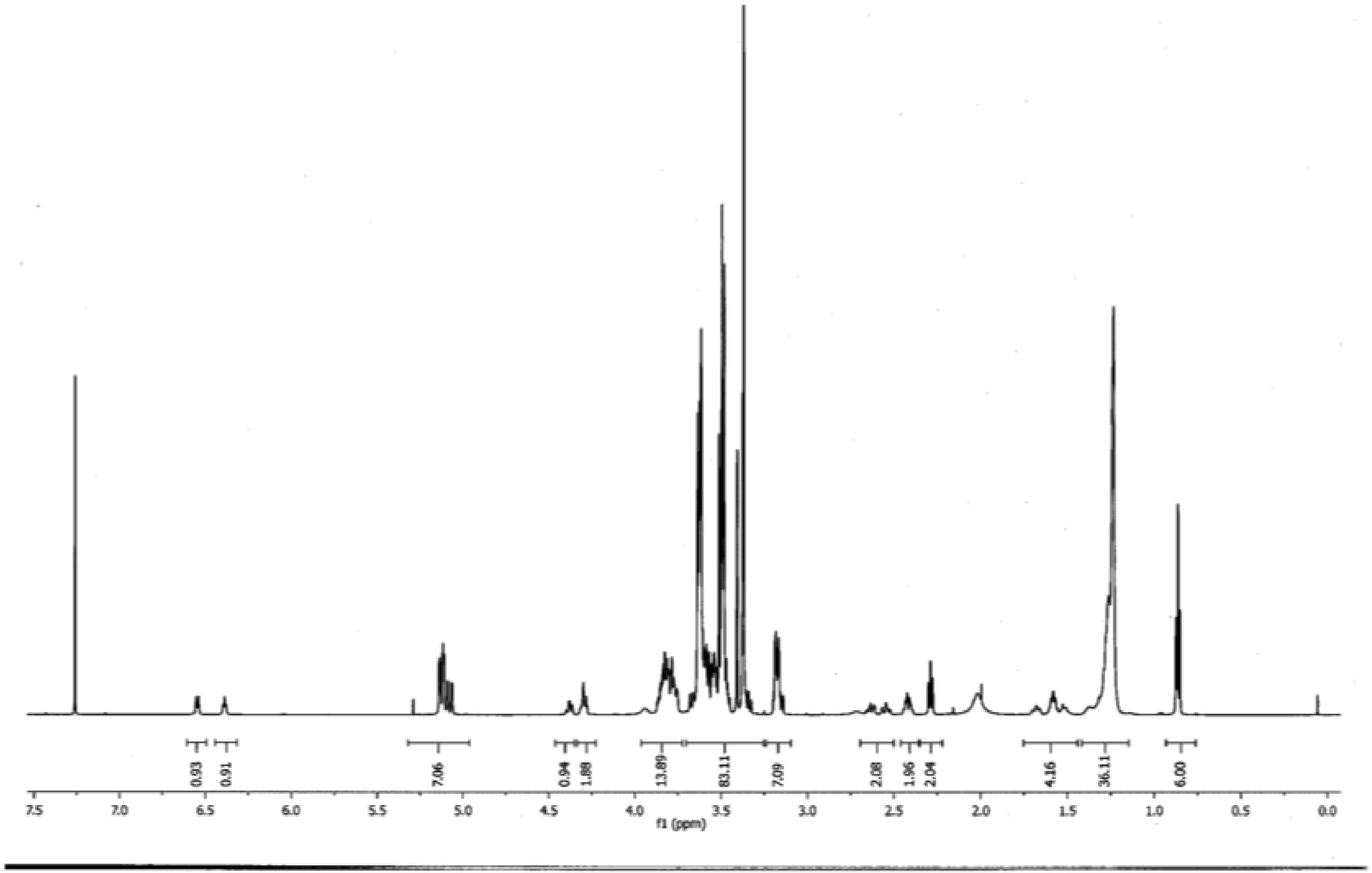

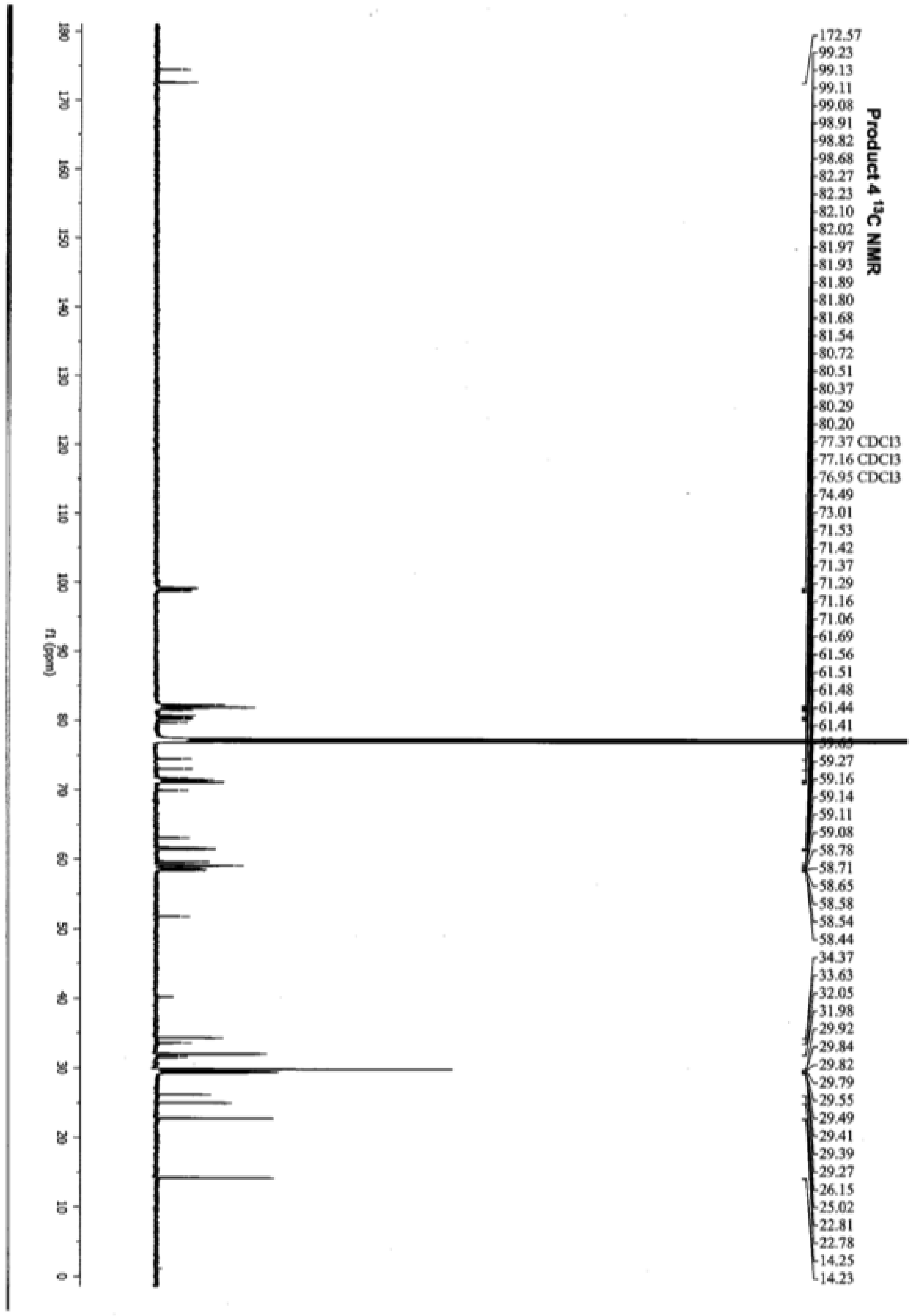
Product 4 13C NMR
172.57
99.23
99.13
99.11
99.08
98.91
98.82
98.68
82.27
82.23
82.10
82.02
81.97
81.93
81.89
81.80
81.68
81.54
80.72
80.51
80.37
80.29
80.20
77.37 CDCl3
77.16 CDCl3
76.95 CDCl3
74.49
73.01
71.53
71.42
71.37
71.29
71.16
71.06
61.69
61.56
61.51
61.48
61.44
61.41
59.27
59.16
59.14
59.11
59.08
58.78
58.71
58.65
58.58
58.54
58.44
34.37
33.63
32.05
31.98
29.92
29.84
29.82
29.79
29.55
29.49
29.41
29.39
29.27
26.15
25.02
22.81
22.78
14.25
14.23
f1 (ppm)

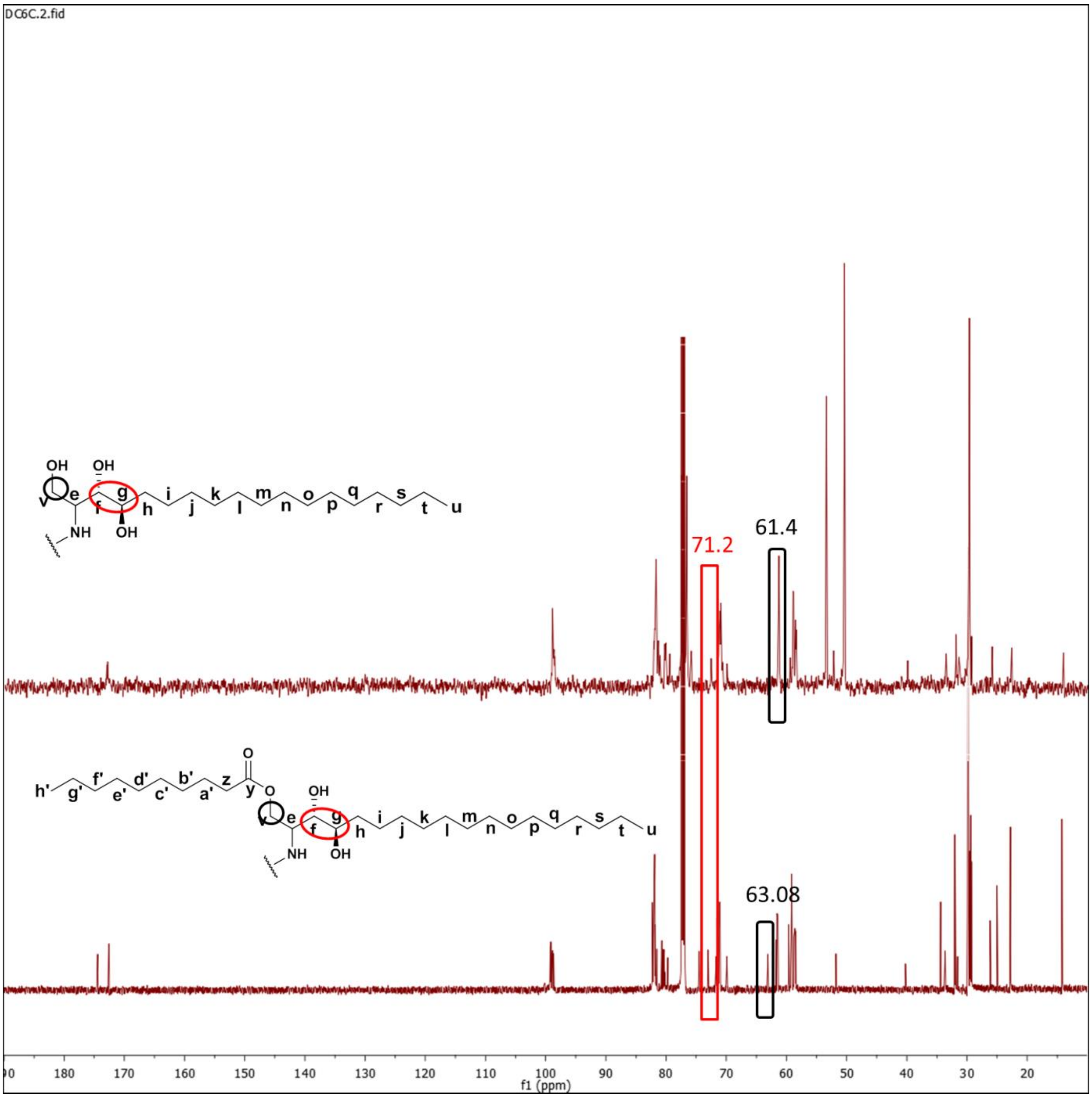
DC6C.2.fid
71.2
61.4
63.08
f1 (ppm)

Shift of ^{13}C chemical shift of CH_2(v) compared to chemical shifts of CH(f,g) showing clearly the regioselectivity of the O-Acylation.

Elemental Composition Report (4) **Page 1**

Single Mass Analysis
Tolerance = 2.0 PPM / DBE: min = -1.5, max = 50.0
Isotope cluster parameters: Separation = 1.0 Abundance = 1.0%

Monoisotopic Mass, Odd and Even Electron Ions
807 formula(e) evaluated with 1 results within limits (up to 50 closest results for each mass)

MYCDS-C10 9 (0.684) AM (Cen,5, 80.00, Ar,5000.0,490.89,1.00,LS 10); Sm (SG, 2x3.00); Cm (9:11) 1: TOF MS ES+ 2.93e3

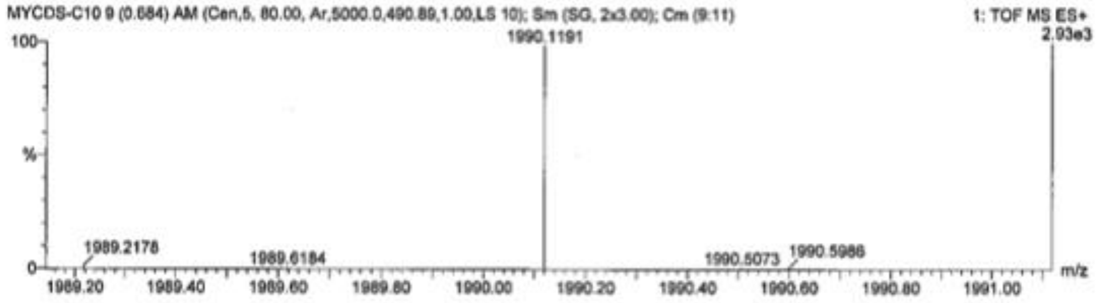

Mass	Calc. Mass	mDa	PPM	DBE	Score	Formula
Minimum:				-1.5		
Maximum:		5.0	2.0	50.0		
1990.1191	1990.1228	-3.7	-1.8	10.5	1	C94 H170 N2 O40 Na

Product 5 ^{1}H NMR

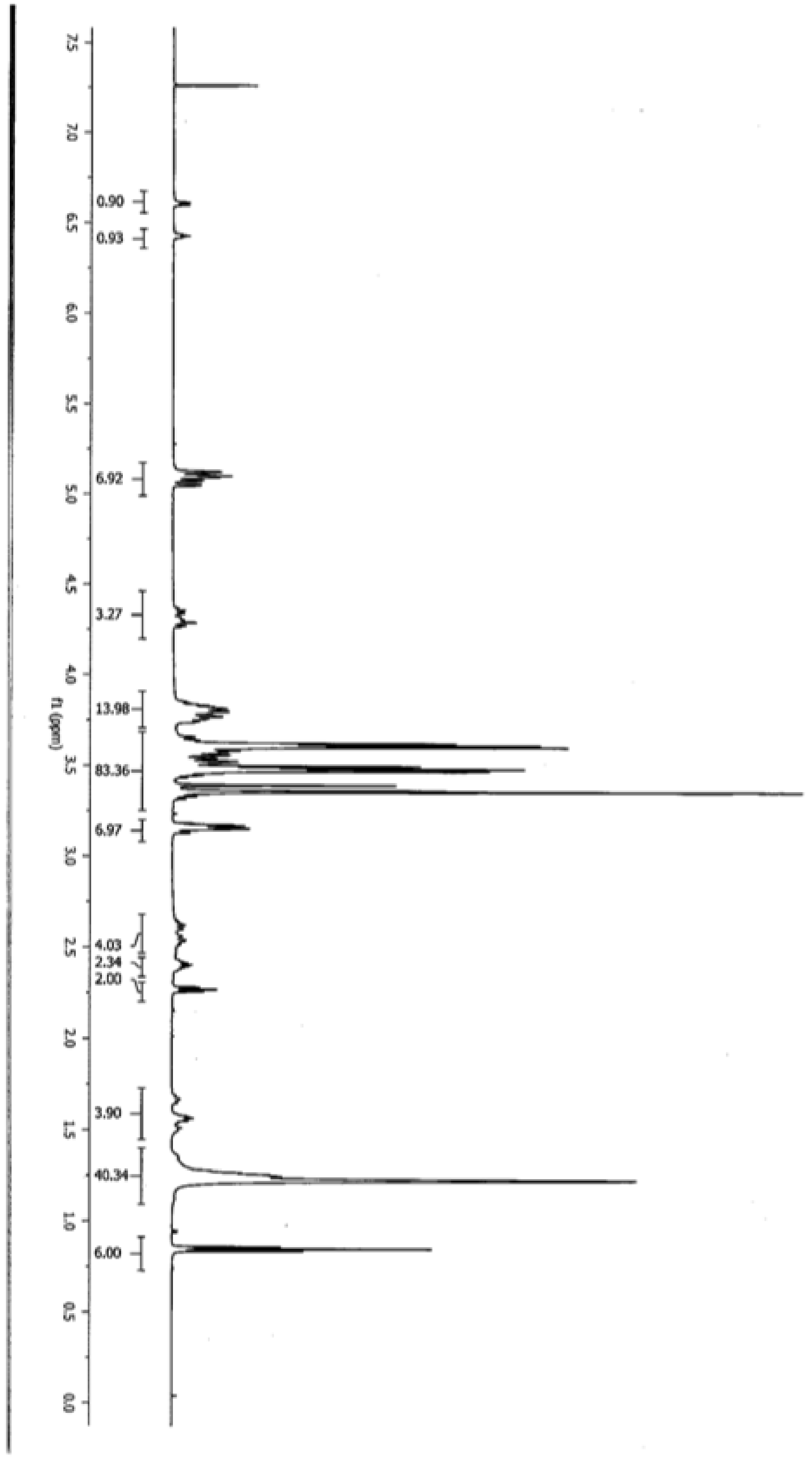

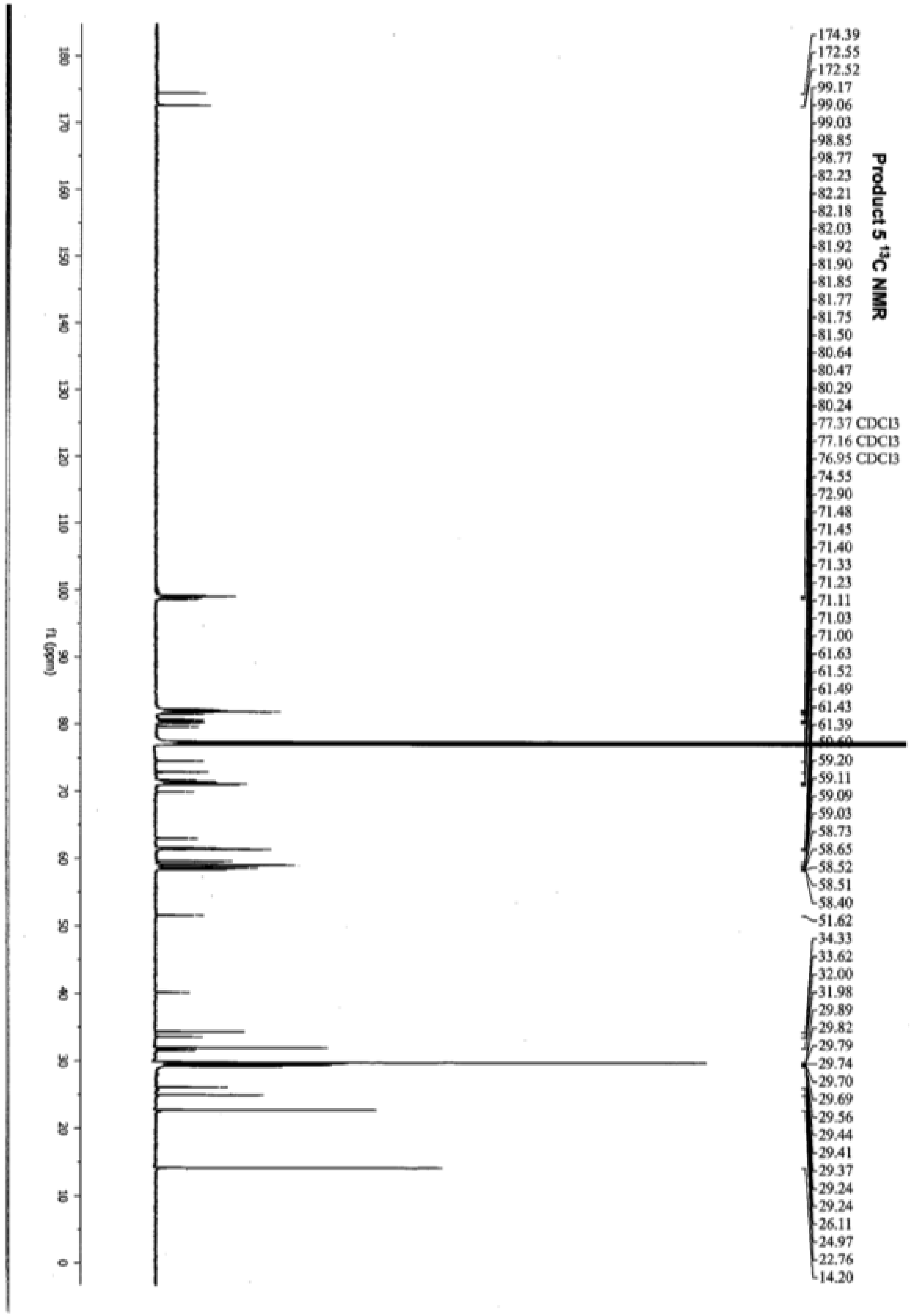
Product 5 13C NMR
174.39
172.55
172.52
99.17
99.06
99.03
98.85
98.77
82.23
82.21
82.18
82.03
81.92
81.90
81.85
81.77
81.75
81.50
80.64
80.47
80.29
80.24
77.37 CDCl3
77.16 CDCl3
76.95 CDCl3
74.55
72.90
71.48
71.45
71.40
71.33
71.23
71.11
71.03
71.00
61.63
61.52
61.49
61.43
61.39
59.20
59.11
59.09
59.03
58.73
58.65
58.52
58.51
58.40
51.62
34.33
33.62
32.00
31.98
29.89
29.82
29.79
29.74
29.70
29.69
29.56
29.44
29.41
29.37
29.24
29.24
26.11
24.97
22.76
14.20
f1 (ppm)

Elemental Composition Report (5)

Single Mass Analysis
Tolerance = 2.0 PPM / DBE: min = -1.5, max = 50.0
Isotope cluster parameters: Separation = 1.0 Abundance = 1.0%

Monoisotopic Mass, Odd and Even Electron Ions
804 formula(e) evaluated with 2 results within limits (up to 50 closest results for each mass)

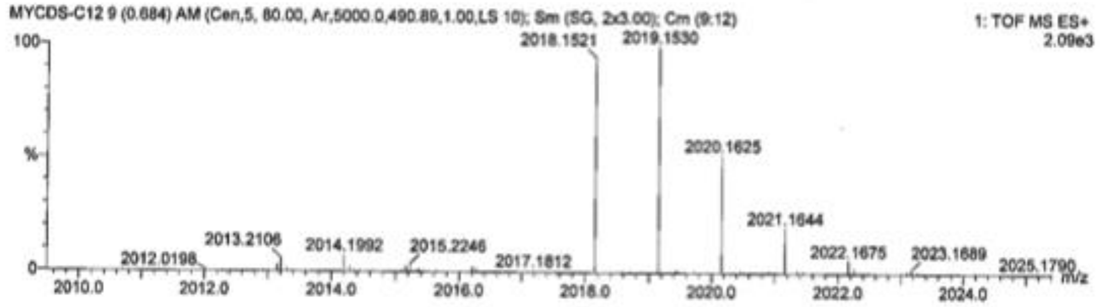

Minimum:				-1.5		
Maximum:		5.0	2.0	50.0		
Mass	Calc. Mass	mDa	PPM	DBE	Score	Formula
2018.1521	2018.1541	-2.0	-1.0	10.5	1	C96 H174 N2 O40 Na
	2018.1554	-3.3	-1.6	21.0	2	C96 H164 N13 O31 Na

Product 6 ^{1}H NMR

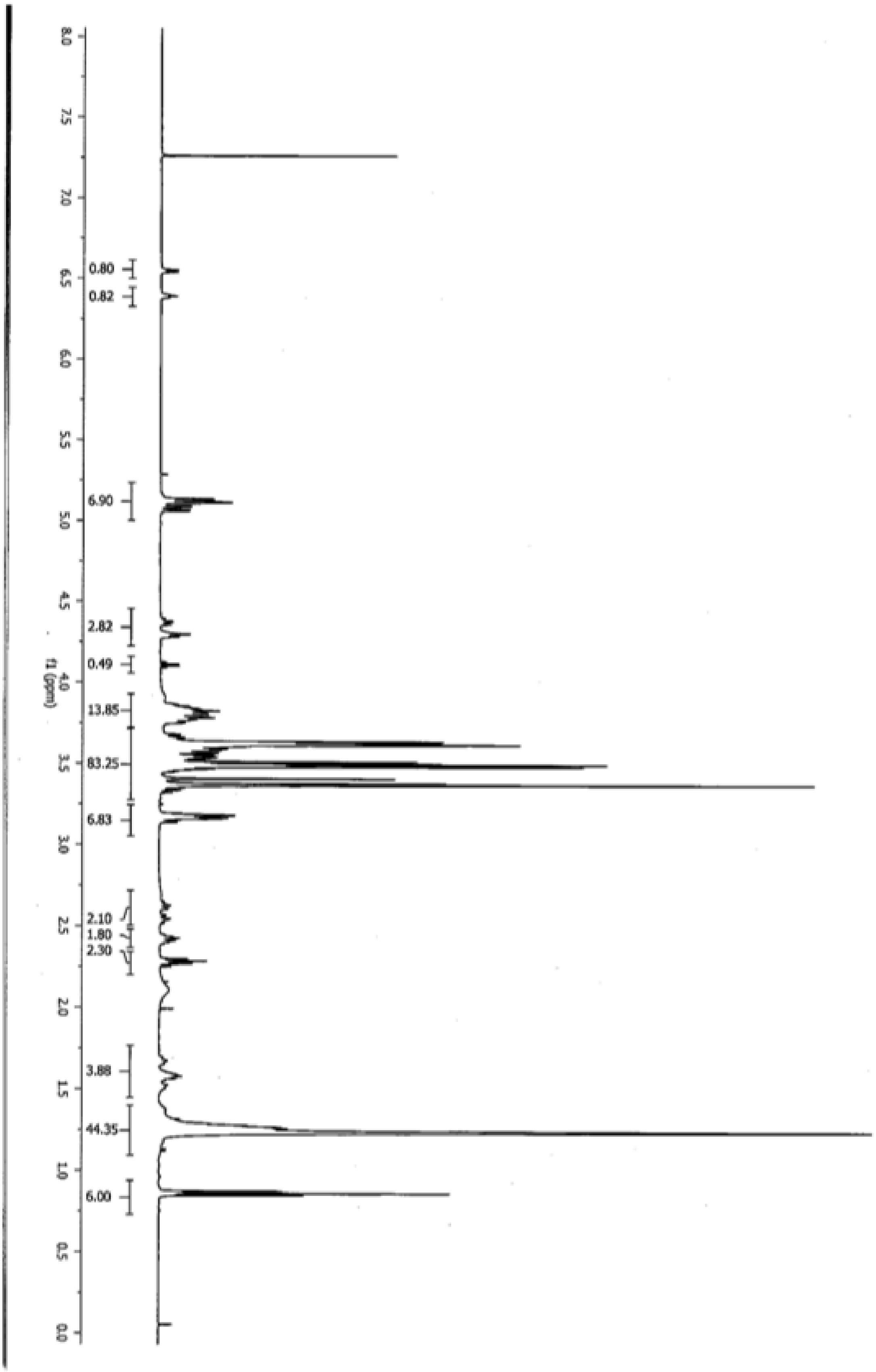

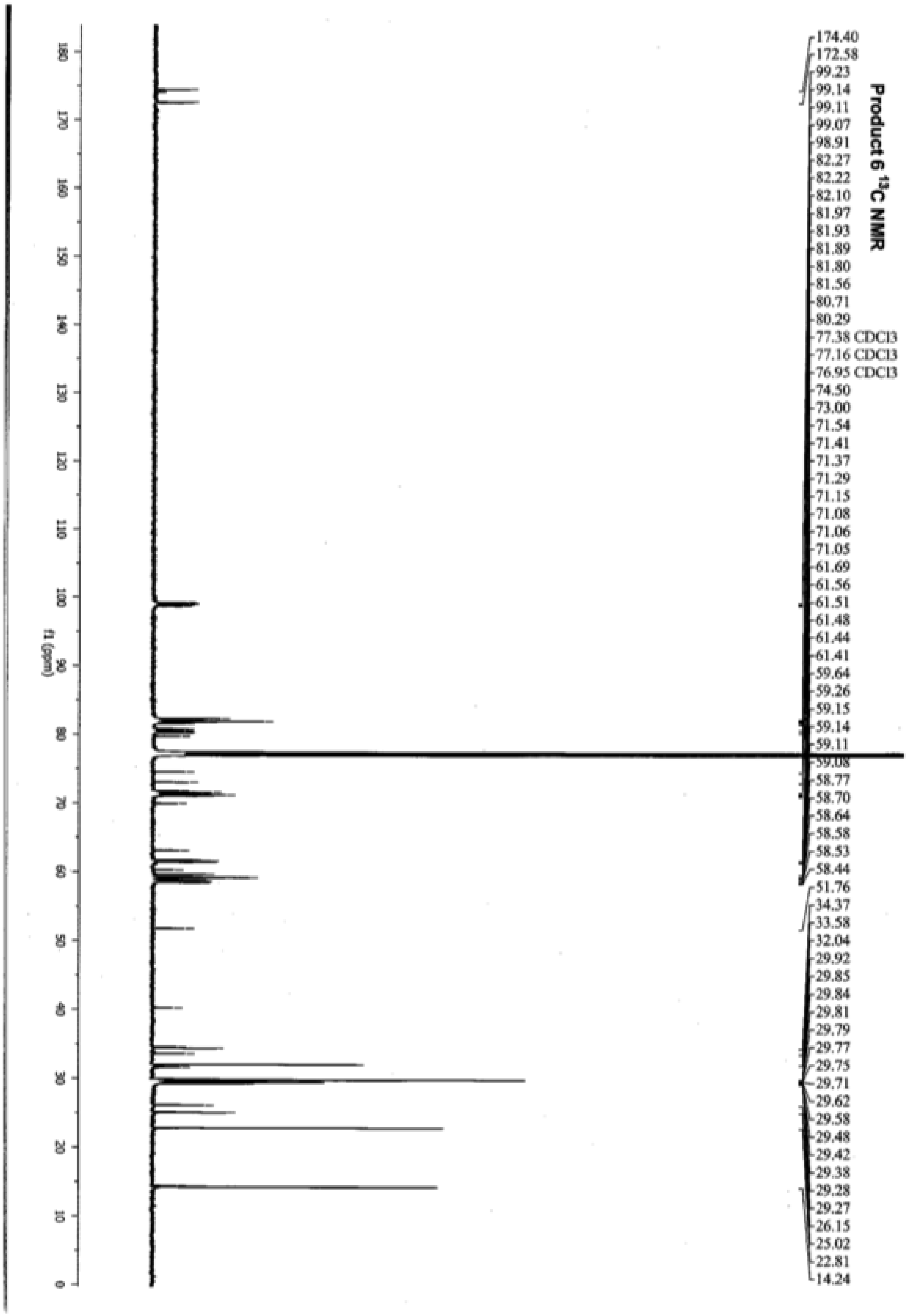
Product 6 ^{13}C NMR
174.40
172.58
99.23
99.14
99.11
99.07
98.91
82.27
82.22
82.10
81.97
81.93
81.89
81.80
81.56
80.71
80.29
77.38 CDCl3
77.16 CDCl3
76.95 CDCl3
74.50
73.00
71.54
71.41
71.37
71.29
71.15
71.08
71.06
71.05
61.69
61.56
61.51
61.48
61.44
61.41
59.64
59.26
59.15
59.14
59.11
59.08
58.77
58.70
58.64
58.58
58.53
58.44
51.76
34.37
33.58
32.04
29.92
29.85
29.84
29.81
29.79
29.77
29.75
29.71
29.62
29.58
29.48
29.42
29.38
29.28
29.27
26.15
25.02
22.81
14.24
f1 (ppm)
180
170
160
150
140
130
120
110
100
90
80
70
60
50
40
30
20
10
0

Elemental Composition Report (6)

Single Mass Analysis
Tolerance = 5.0 PPM / DBE: min = -1.5, max = 50.0
Isotope cluster parameters: Separation = 1.0 Abundance = 1.0%

Monoisotopic Mass, Odd and Even Electron Ions
798 formula(e) evaluated with 2 results within limits (up to 50 closest results for each mass)

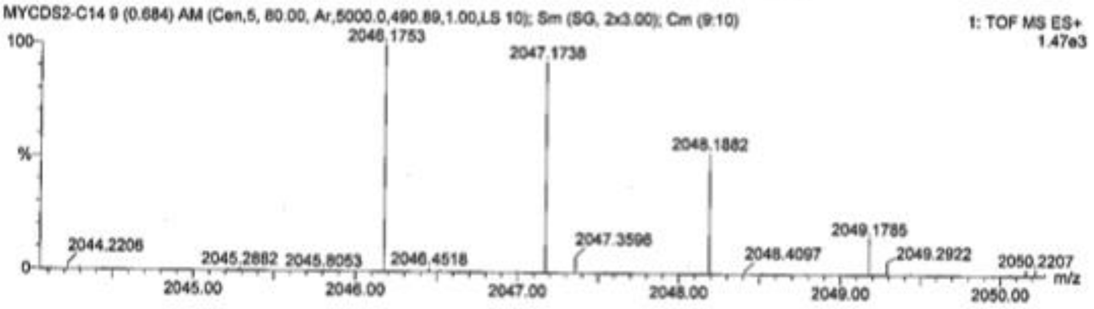

Minimum:				-1.5		
Maximum:		5.0	5.0	50.0		
Mass	Calc. Mass	mDa	PPM	DBE	Score	Formula
2046.1753	2046.1741	1.2	0.6	16.0	2	C98 H172 N7 O36 Na
	2046.1854	-10.1	-4.9	10.5	1	C98 H178 N2 O40 Na

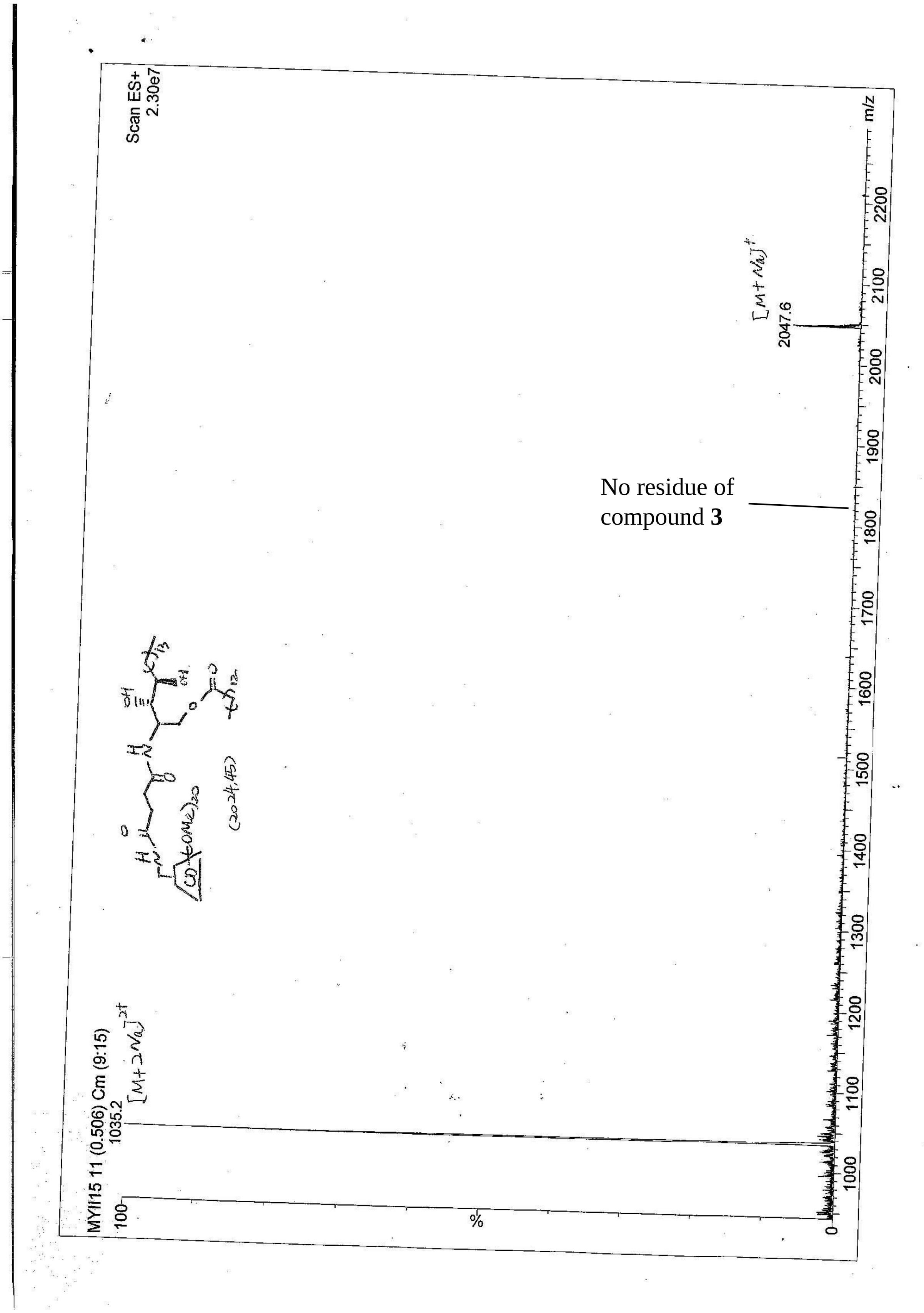
MYII15 11 (0.506) Cm (9:15)
Scan ES+
2.30e7
1035.2
[M+2Na]2+
2047.6
[M+Na]+
No residue of compound **3**
%
m/z

Product 7 ^{1}H NMR

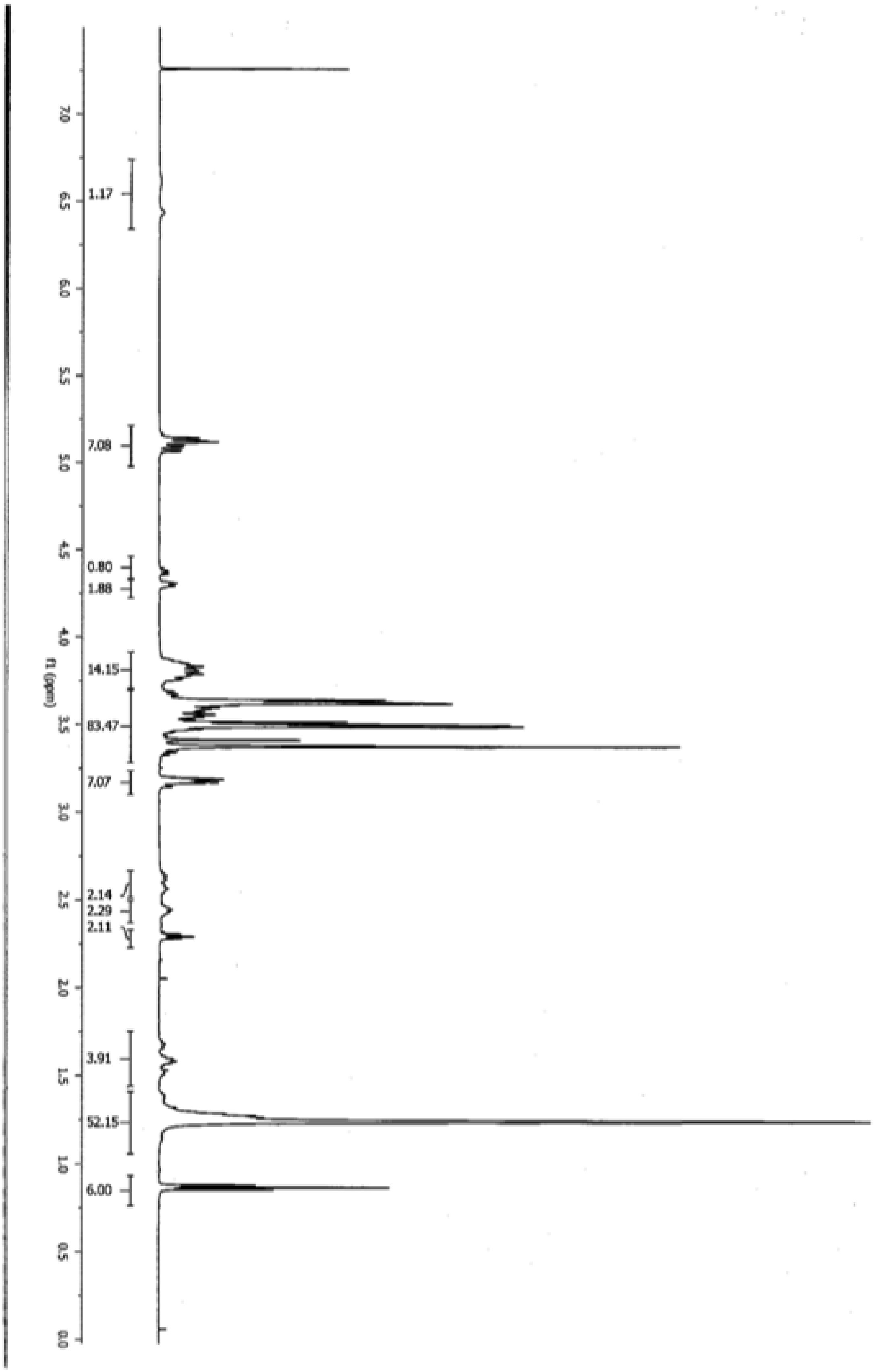

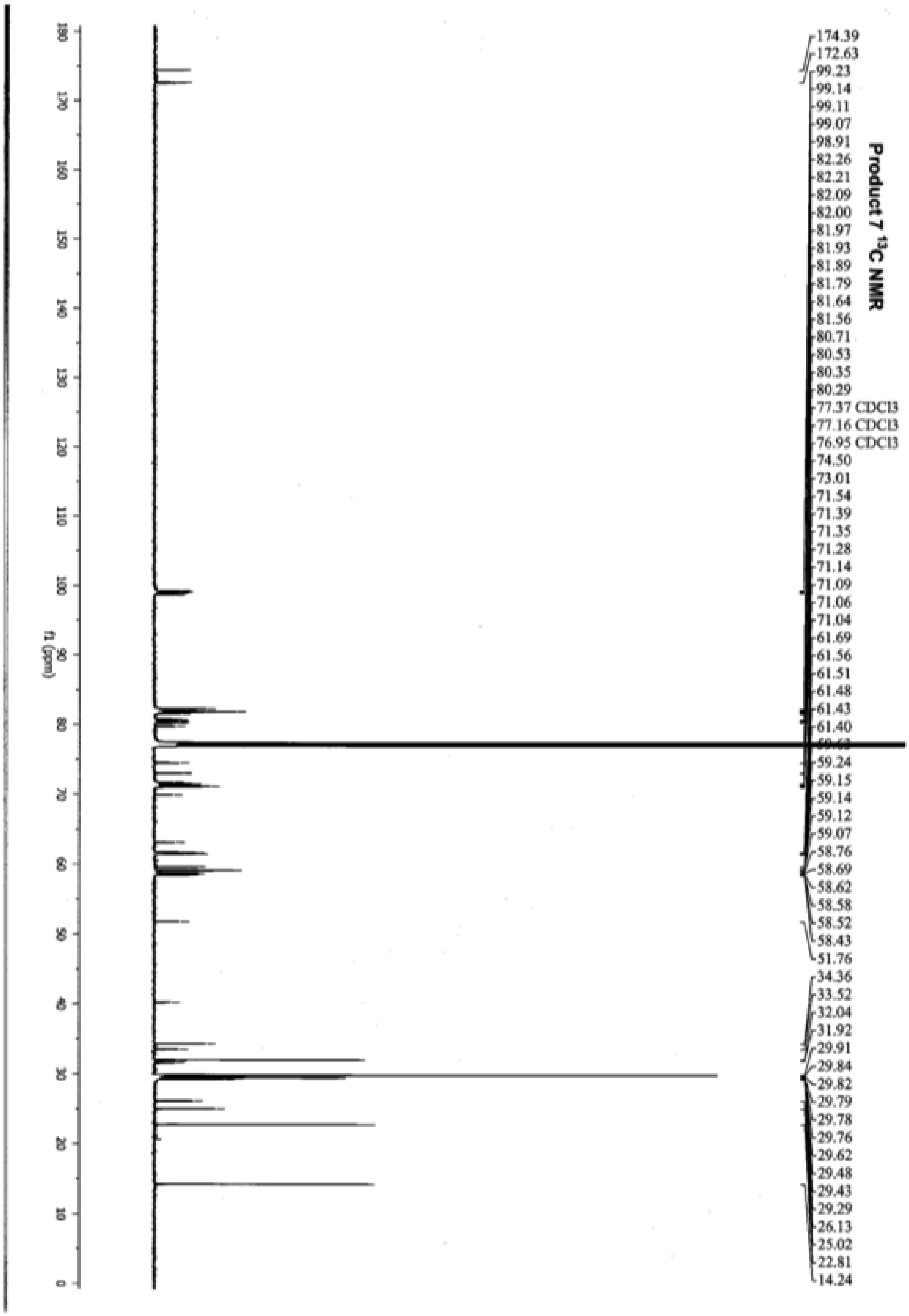
Product 7 13C NMR
174.39
172.63
99.23
99.14
99.11
99.07
98.91
82.26
82.21
82.09
82.00
81.97
81.93
81.89
81.79
81.64
81.56
80.71
80.53
80.35
80.29
77.37 CDCl3
77.16 CDCl3
76.95 CDCl3
74.50
73.01
71.54
71.39
71.35
71.28
71.14
71.09
71.06
71.04
61.69
61.56
61.51
61.48
61.43
61.40
59.24
59.15
59.14
59.12
59.07
58.76
58.69
58.62
58.58
58.52
58.43
51.76
34.36
33.52
32.04
31.92
29.91
29.84
29.82
29.79
29.78
29.76
29.62
29.48
29.43
29.29
26.13
25.02
22.81
14.24
f1 (ppm)

Elemental Composition Report

Single Mass Analysis
Tolerance = 5.0 PPM / DBE: min = -1.5, max = 50.0
Isotope cluster parameters: Separation = 1.0 Abundance = 1.0%

Monoisotopic Mass, Odd and Even Electron Ions
792 formula(e) evaluated with 3 results within limits (up to 50 closest results for each mass)

MYCDS-C18 8 (0.608) AM (Cen,5, 80.00, Ar,5000.0,490.89,1.00,LS 10); Sm (SG, 2x3.00); Cm (8:10)   1: TOF MS ES+ 1.30e3

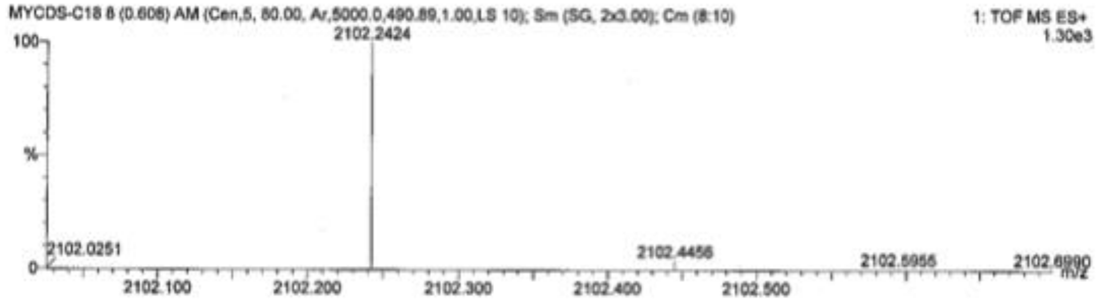

Mass	Calc. Mass	mDa	PPM	DBE	Score	Formula
Minimum:				-1.5		
Maximum:		5.0	5.0	50.0		
2102.2424	2102.2480	-5.6	-2.6	10.5	3	C102 H186 N2 O40 Na
	2102.2367	5.7	2.7	16.0	2	C102 H180 N7 O36 Na
	2102.2493	-6.9	-3.3	21.0	1	C102 H176 N13 O31 Na
